# Classification of user queries according to a hierarchical medical procedure encoding system using an ensemble classifier

**DOI:** 10.3389/frai.2022.1000283

**Published:** 2022-11-04

**Authors:** Yihan Deng, Kerstin Denecke

**Affiliations:** Department of Technology and Computer Science, Institute for Medical Informatics, Bern University of Applied Sciences, Biel/Bienne, Switzerland

**Keywords:** ensemble classifier, CHOP, hierarchical classification, medical procedure, feature selection

## Abstract

The Swiss classification of surgical interventions (CHOP) has to be used in daily practice by physicians to classify clinical procedures. Its purpose is to encode the delivered healthcare services for the sake of quality assurance and billing. For encoding a procedure, a code of a maximal of 6-digits has to be selected from the classification system, which is currently realized by a rule-based system composed of encoding experts and a manual search in the CHOP catalog. In this paper, we will investigate the possibility of automatic CHOP code generation based on a short query to enable automatic support of manual classification. The wide and deep hierarchy of CHOP and the differences between text used in queries and catalog descriptions are two apparent obstacles for training and deploying a learning-based algorithm. Because of these challenges, there is a need for an appropriate classification approach. We evaluate different strategies (multi-class non-terminal and per-node classifications) with different configurations so that a flexible modular solution with high accuracy and efficiency can be provided. The results clearly show that the per-node binary classification outperforms the non-terminal multi-class classification with an F1-micro measure between 92.6 and 94%. The hierarchical prediction based on per-node binary classifiers achieved a high exact match by the single code assignment on the 5-fold cross-validation. In conclusion, the hierarchical context from the CHOP encoding can be employed by both classifier training and representation learning. The hierarchical features have all shown improvement in the classification performances under different configurations, respectively: the stacked autoencoder and training examples aggregation using true path rules as well as the unified vocabulary space have largely increased the utility of hierarchical features. Additionally, the threshold adaption through Bayesian aggregation has largely increased the vertical reachability of the per node classification. All the trainable nodes can be triggered after the threshold adaption, while the F1 measures at code levels 3–6 have been increased from 6 to 89% after the threshold adaption.

## 1. Introduction

The Swiss classification of surgical interventions (CHOP) (BFS, 2022) has to be used in daily practice by physicians to classify clinical procedures. Its purpose is to encode the delivered healthcare services for the sake of quality assurance and billing. It supports the cost reimbursement between hospitals and insurance companies as well as the reporting to healthcare authorities. For encoding a procedure, a code of a maximal of 6-digits has to be selected from the classification system. For instance, the code “00.65.11” refers to the free textual category description “Perkutanes transluminales Einsetzen von intrakraniellen vaskulären Mikrostent(s)” (translation follows). The first two digits “00” indicate the basic code assignment at the chapter level (interventions not specified elsewhere). The additional two digits “65” narrow down the target procedure to a relatively specific category (“insertion of other types of intracranial vascular stents”). The last two digits “11” lead the classification subsequently to a more concrete type of insertion of vascular micro stents. The CHOP comprises more than 14,000 different classes at six levels. 45% of the non-terminal labels have more than five sub-nodes.

Currently, the classification of clinical procedures is realized in practice by a rule-based system composed of encoding experts and a manual search in the CHOP catalog. The quality of encoding is, therefore, strongly influenced by the quality of handcrafted rules and the competence of the human encoder. In the meantime, most of the work in research has focused on the evaluation of end-to-end neural network training based on clinical shared data in English like MIMIC II (Lee et al., 2011) and MIMIC III (Johnson et al., 2016). A suitable deep learning based method for German clinical procedure encoding has not been well explored. In this paper, we will investigate the possibility of automatic CHOP code generation based on a short query. The wide and deep hierarchy of CHOP and the differences between text used in queries and catalog descriptions are two apparent obstacles for training and deploying a learning-based algorithm. Because of these challenges, there is a need for an appropriate classification approach. We will evaluate different strategies (multi-class non-terminal and per-node classifications) with different configurations so that a flexible modular solution with high accuracy and efficiency can be provided. Beyond that, the log data for the German OPS classification (BfArM, 2022) (German clinical procedure encoding corresponding to Swiss CHOP) will be employed to extend the CHOP log set based on category matching, the classification result based on different German data sets and networks configurations will be evaluated within real industrial settings.

## 2. Problem definition

The code assignment for a CHOP category is a task of multilabel classification. Each query will be assigned a sequence of correlated CHOP codes from top-level chapters to specific subcategories at lower levels. Exploring the complex hierarchy of CHOP encoding (labels) is relevant for the design of a holistic solution for multilabel assignment. Notably, we need to concentrate on the analysis and use of features learned from the CHOP hierarchy to facilitate the classification of single nodes and the prediction of multilabel sequences along the related CHOP node path.

Formally, let a CHOP category be a partial order for medical procedure *MP*, which is presented in a tree-structured category hierarchy *h* over an ordered set (*P*_*h*_, ≺). *P*_*h*_ is the category set of 6-digit hierarchical procedure encoding *MP*, which has been introduced in Section 1. The *i, j*, and *k* represent three related categories within the hierarchy *MP*. The ≺ represents the is-a relationship between two categories (Wu et al., 2005). The is-a relation is first irreversible:


(1) $$\begin{array}{l} \text{if }P_{i}≺P_{j}\text{ then}\forall P_{i},P_{j}\in MP,p_{j}⊀p_{i} \end{array}$$

Furthermore, the relationship is both anti-reflective and transitive (Silla and Freitas, 2011).


(2) $$\forall P_{i}\;\in MP,P_{i}⊀P_{i}$$

(3) $$\forall P_{i},P_{j},P_{k}\;\in MP,P_{i}≺P_{j}\text{ and }P_{j}≺P_{k}\text{ imply }P_{i}≺P_{k}$$

The hierarchy of the medical procedure follows the true path rule (Valentini, 2009) along the parent-child chains. It means if a query has been annotated with a specific class label, then it is annotated with all its parent classes recursively. If one query does not belong to a class, it does not belong to all of its offspring classes. The three features mentioned earlier of the CHOP category cause the following challenges to be considered when designing a method for code generation:

1. **Training of classifiers considering hierarchy information:** How should the hierarchical features be incorporated in the training of classifiers?
2. **Feature selection:** How can we employ the features of hierarchical structures by representation learning? How can we select the most representative feature set for different categories based on the hierarchical context?
3. **Methods of hierarchical prediction:** Is it possible to define a threshold for each class separately and dynamically? How should the prediction threshold be determined based on the output of the base classifier?

## 3. Hierarchical evaluation metrics

Unlike conventional evaluation metrics based on the comparison between one predicted label and a single label from the benchmark, hierarchical evaluation metrics are calculated using the entire predicted path. The predicted label path will be compared to the entire label path to the root. The original label *Y* and its ancestors (*y*_1_, …, *y*_*n*_) on the true paths *Y*_path_ can be represented as:


(4) $$\begin{array}{l} Y_{\text{path}}=Y\cup \text{An}\left(y_{1}\right)\cup \text{An}\left(y_{2}\right)\cup \cdots \cup \mathrm{An}\left(y_{n}\right) \end{array}$$

where (*y*_1_, …, *y*_*n*_) includes the benchmark y and all its n ancestors (An) correspond to the sample X. The predicted labels within the true path can be represented as:


(5) $$\begin{array}{l} \hat{Y_{\text{path}}}=\hat{Y}\cup \mathrm{An}\left(\hat{y_{1}}\right)\cup \mathrm{An}\left(\hat{y_{2}}\right)\cup \cdots \cup \mathrm{An}\left(\hat{y_{m}}\right) \end{array}$$

where the (*y*_1_, …, *y*_*m*_) indicates the predicted label ŷ and all its m ancestors under the input of sample X. The near loss or distance between the predicted path and labeled paths is calculated based on the total steps $\underset{\text{E}}{\sum }\left(Y,\hat{Y}\right)$ between predicted y and label y, where $\underset{\text{E}}{\sum }\left(a,b\right)$ indicates the total steps between a and b.


(6) $$\begin{array}{l} \Gamma_{\text{Distance}}\left(Y,\hat{Y}\right)=\underset{\text{E}}{\sum }\left(Y,\hat{Y}\right) \end{array}$$

The hierarchical precision (Hp) and recall (Hr) are metrics for the evaluation of hierarchical classification methods. Hp indicates the value of the intersection of prediction and original label divided by the prediction set size. Hr represents the intersection of prediction and labels divided by the original labels. The hierarchical metrics of precision and recall reflect a hypo-tactic projection (is-a) between the ancestor nodes and descendant nodes. They show the correlations between general categories and specific categories and in this way implicitly, represent the depth of miss classification and their influence on the entire true path.


(7) $$\begin{array}{l} Precision_{H}=\frac{\left|\hat{Y_{\text{path}}}\cap Y_{\text{path}}\right|}{\left|\hat{Y_{\text{path}}}\right|} \end{array}$$

(8) $$\begin{array}{l} Recall_{H}=\frac{\left|Y_{\text{path}}\cap \hat{Y_{\text{path}}}\right|}{\left|Y_{\text{path}}\right|} \end{array}$$

## 4. Related studies

Early approaches of clinical encoding have been implemented as rule-based classification (Farkas and Szarvas, 2008), or feature engineering with conventional machine learning (Medori and Fairon, 2010). As features, uni-grams and bi-grams (Chute et al., 2006), syntactic features (Goldstein et al., 2007), or similarity scores obtained through concept mapping with external knowledge bases (Atutxa et al., 2018) were applied to train the encoding algorithms. Different conventional classifiers like Na$\ddot{ı}$ve Bayes (Chute et al., 2006), C4.5 decision tree (Farkas and Szarvas, 2008), SVM (Perotte et al., 2013), and random forest (Atutxa et al., 2018) have been confirmed to be capable of assigning a category code to text input for different medical encoding systems.

Pérez et al. (2018) proposed an encoding mechanism based on a transformer architecture. CNN and RNN encoding and their combinations have been evaluated. With the further development of neural networks, the representation obtained through word embedding and gated recurrent unit (GRU) has also been considered for the task of ICD-9 classification using discharge summaries from the MIMIC III database (Catling et al., 2018). The prediction based on neural models has achieved the best F1 score of 68.8% at the chapter level among the entire ICD-9 code hierarchy. Using 1D Convolution, LSTM, and GRU as prediction models and word sequence embedding as input, Huang et al. (2018) achieved nearly 90% accuracy on the task of classification of the top-10 ICD-9 codes. In order to learn a suitable representation for the enriched non-sequential clinical term vectors, Deng et al. (2018) employed stacked denoising autoencoders to learn a reduced vector representation for the CHOP catalog and queries. In particular, the representations were firstly learned locally and then fine-tuned globally with a supervised training set. The obtained representation achieved a Micro F1 of 70.78% on the task of query and catalog matching. Moreover, the classifier trained on hierarchical features regarding the medical encoding outperformed the flat classification based on a single code training set (Perotte et al., 2013; Catling et al., 2018). Valentini (2011) applied hierarchical ensembles for gene function prediction in Gene Ontology and FunCat taxonomies. The true path rule (positive predictions of a node influence ancestors, while negative predictions influence offspring) can also be applied to the CHOP classification (Valentini, 2009) since it also has a tree-like category structure.

In contrast to existing work, we will conduct the hierarchical classification with a hierarchy of base classifiers. These base classifiers are trained with a large number of real user queries. The text representation will be learned using stacked denoising autoencoders. The hierarchical training set will be gathered through the rolling up of a descendent training set to reflect the nature of CHOP encoding. As state of the art described before indicates, the automatic code assignment in the entire code system can typically provide only accuracy around 30–60% (Perotte et al., 2013; Catling et al., 2018), while the classification based on a selected subset of the full encoding system can reach a higher accuracy (90%) (Boytcheva, 2011; Atutxa et al., 2018). Therefore, we will conduct the code assignment on a set of the most frequently used CHOP codes as classification targets.

Cao et al. (2020) exploited hyperbolic and Co-graph to represent the code co-occurrence and hierarchical correlation. With the two separate representation enhancements, they have achieved the state of the art accuracy using testing data from MIMIC III (top 50), MIMIC II, and MIMIC III all labels. Xun et al. (2020) have used Correlation Networks for Extreme Multi-label Text Classification with examples of ICD classification. One additional correlation detection layer has been added above the prediction and learning layer so that the correlation between the code labels can be modeled better. Song et al. (2020) have applied graphic neural networks (GCNN and GRNN) to label description to encode the label affinity to the model. However, the neural network based methods (Cao et al., 2020; Song et al., 2020; Xun et al., 2020) have only focused on the end-to-end model implementation for automatic ICD encoding, the concrete pro node situation and model explainability have largely been ignored. Moreover, the models have only been validated on the MIMIC II and MIMIC III data sets with limited label selection. Transferring the models to a real medical data set with clinical validation and to other languages requires still further verification.

## 5. Data set

As underlying data set, we have gathered the queries from server logs and bug tracker entries from a real production server of a current rule-based code retrieval system (refer to Table 1). In the following part of this paper, by “query,” we are referring to search phrases from the bug tracker or server logs. The training set is highly imbalanced, i.e., the number of negative training examples is clearly larger than the number of positive examples. Since there is no interconnection between categories, the CHOP category can be transformed into a tree structure. Each parent node has a certain number of children nodes, whereas each child node is linked to only one direct parent node. The CHOP-tree comprises six levels, which reflect the six hierarchical levels of the CHOP. To incorporate the hierarchical nature of the CHOP structure into the training set, we have applied a “siblings” policy mentioned in Fagni and Sebastiani (2007); Silla and Freitas (2011): The positive examples for a code c are used as positive examples for its ancestors f. The positive examples for a code c are used as negative examples for its siblings s. If this positive example for a sibling s of code c occurs as a positive example for c, then this positive example will not be used as a negative example for c.

**Table 1:** Training data statistics of CHOP and OPS+CHOP.

**Metrics**	**CHOP**	**OPS+CHOP**
Level 1	1,784,645	4,874,823
Level 2	887,163	2,229,208
Level 3	1,048,608	2,733,439
Level 4	702,454	1,684,298
Level 5	408,901	1,119,466
Level 6	80,415	177,763
Total	4,912,186	12,818,997
Query count	55,845	91,999
Vocabularies	46,169	53,120
Trainable nodes	2,793	3,545

The training pairs at each level are listed. The query count indicates the sum of unique user queries from all query pairs. Vocabulary represents the total number of unique words in CHOP and OPS+CHOP. We selected the CHOP nodes with at least 21 positive examples as trainable nodes for data balancing.

In the data set, we could observe that there are CHOP categories that provide a large number of server logs from the current system. These are categories that are frequently used in daily clinical work. On the other hand, rarely used procedures in daily practice result in a very small number of logs for the corresponding CHOP category, or no log data is available. Hence, we decided to focus on the most frequently used CHOP categories to develop a CHOP encoding classifier with reliable accuracy for the most frequently used CHOP codes. With this restriction, we obtained a node set with 2,793 most frequently used CHOP categories from all six hierarchical levels. Seven hundred of them are non-terminal codes, while the rest 2,093 categories are leaf nodes. We have 4,912,180 training pairs (505,022 positive and 4,407,158 negative) from these three different sources in the form of “*query, code id, label*.”

**Figure 1:**
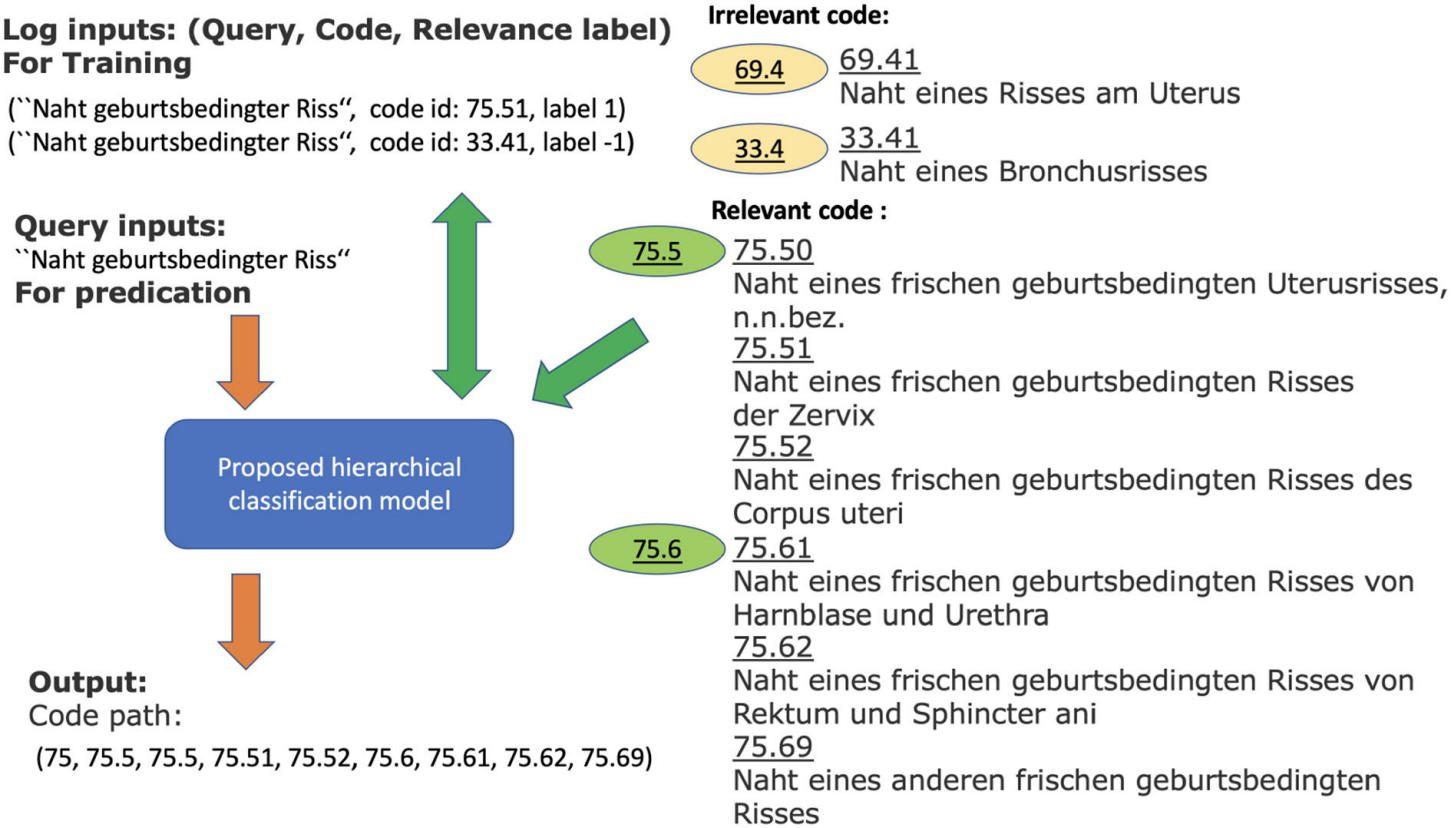
Working example for query log “Naht geburtsbedingter Riss” and relevant and irrelevant codes within the CHOP. The model with log input generates a list of relevant codes as output. The green codes are relevant (positive examples) to the query, while the brown codes are irrelevant categories (negative examples). The obtained classification model should be able to determine the corresponding category and generate the relevant CHOP code for a query.

To increase the number of training examples, we consider the German medical procedure classification OPS (Operationen- und Prozedurenschluessel). It covers nearly all the CHOP categories. Therefore, the log data from OPS can directly be used as training samples for the corresponding CHOP categories. In order to increase the positive examples for CHOP categories, we have gathered the additional log samples from the encoding system for the German medical procedure classification OPS. The total query-node pairs have been increased from 4,912,186 to 12,818,997. The trainable categories (nodes) have been increased from 2,793 to 3,545 (refer to Table 1). The effectiveness of the transfer training will be evaluated in the following sections. Figure 1 shows a working example of a system log from the current encoding server. The input of the model training is one query log presented with the following tuple: [“Naht geburtsbedingter Riss” (suture of a birth-related tear), code id: 75.51, label 1]. It indicates that the query text belongs to code category 75.51. Label 1 indicates the positive relevance between the query and code, while the query (“Naht geburtsbedingter Riss,” code id 33.4, label –1) indicates that the query is irrelevant to the category with code id 33.4 [“naht eines bronchusrisses” (suture of a bronchus tear), refer to Figure 1].

## 6. Methodology

### 6.1. Overview of classification architecture and strategies

**Figure 2:**
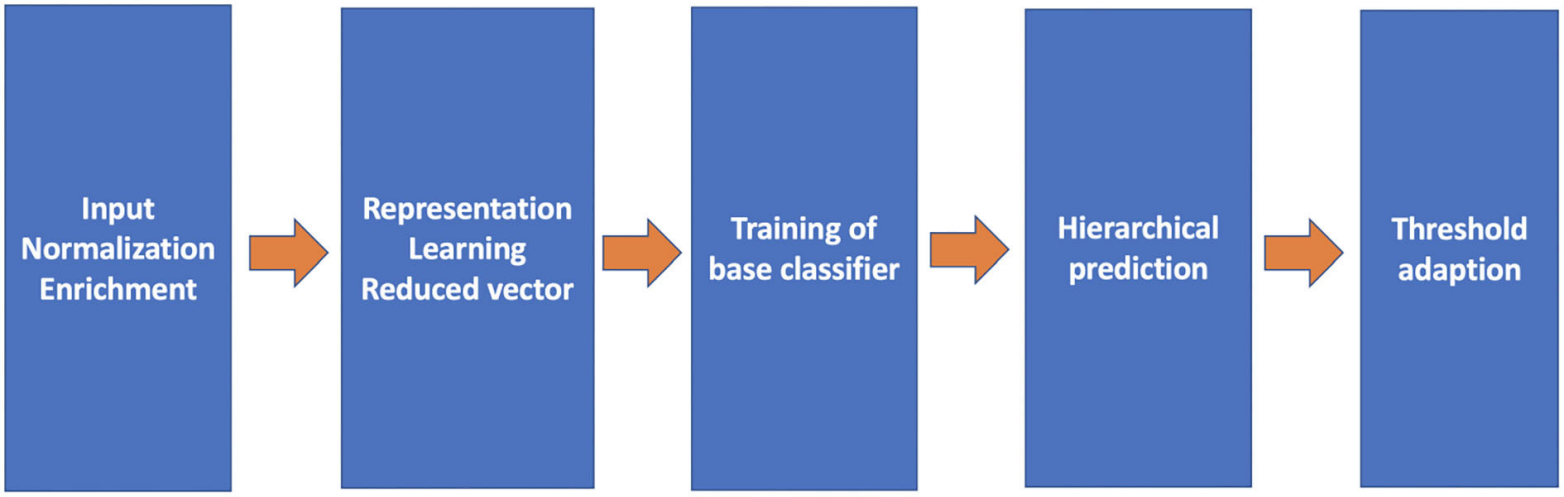
The architecture of the proposed code assigning pipeline.

The proposed pipeline consists of five crucial components (refer to Figure 2): query input normalization and enrichment, representation learning, base classifiers, the hierarchical prediction based on the result of the base classifiers, and the threshold adaption. With these components, three variations of representation learning will be compared.

First, the stacked denoising autoencoder proposed in our previous settings (Deng et al., 2018) is compared to a self-attentive denoising autoencoder. The hierarchical context is concatenated using all possible positive query examples on the same true path.

Second, the classification strategies of multiclass classifiers for non-terminal nodes will be compared with the configuration of assembled binary classifiers (per-node) with a one vs. rest strategy. The multiclass classifier of a non-terminal node is solely applied to determine whether an incoming query is the instance of one of the children nodes. The assembled per-node binary classification follows the strategy of one vs. rest, which means the binary classifier is trained to distinguish the target code from the non-target code. In this sense, the per-node model from one base classifier always has two classes as output, namely relevant to the node or irrelevant to the node, while the multiclass classification has n+1 classes. In contrast, n indicates the number of children nodes and 1 represents the residual category indicating the irrelevance to all other children classes. As input data, the gold standard data from system logs have been separated into a 60% training set and a 40% validation set for 5-fold validation.

The third configuration is the fine-tuning of prediction with thresholds. The post adaption during the prediction will be evaluated based on two algorithms: average bottom-up and Bayesian aggregation. In the following sections, the architectures and configurations will be explained in detail.

### 6.2. Representation learning using autoencoders and self-attention

In contrast to the representation learning based on both query and category text, for the task of query matching, we will learn our representation only using queries from different server logs. The length of the queries in our data set ranges from 2 to 6 terms. To deal with this situation of short phrases, the queries are normalized with a pre-processing toolkit ID MACS^®^. This tool transforms a query string into a bag of concepts model with concepts from the underlying terminology server. The terminology server ID MACS^®^—medical semantic network, software provided by the German company ID Information und Dokumenation im Gesundheitswesen. With ID MACS^®^ it is possible to analyze medical texts and, for instance, to extract structured information on diagnoses and procedures and map them onto a chosen medical terminology (Faulstich et al., 2010; Kreuzthaler et al., 2011). The semantic network incorporates the Wingert Nomenclature, a German derivative of an early version of SNOMED, as a knowledge base (Wingert, 1984). Through this transformation process, the query text has been matched to the medical concepts and depicts the relationship between individual concepts. This bag of concepts model is then enriched by adding further entries for similar medical concepts, weighted by their similarity to the concepts in the original semantic representation. This transformation aims to bridge the syntactic and semantic discrepancy between query and catalog text and to achieve a common language space. This semantic enrichment using similar terms from the ID MACS^®^ results in a 46,170-dimensional sparse vector representation for each text document. We have 55,845 unique query and catalog text pairs resulting in 4,912,180 pairs of matching between enriched semantic representations of documents (queries and catalog texts) and CHOP codes. However, in this bag of concept representation, all information on word order as provided in the original text sequence is lost.

**Figure 3:**
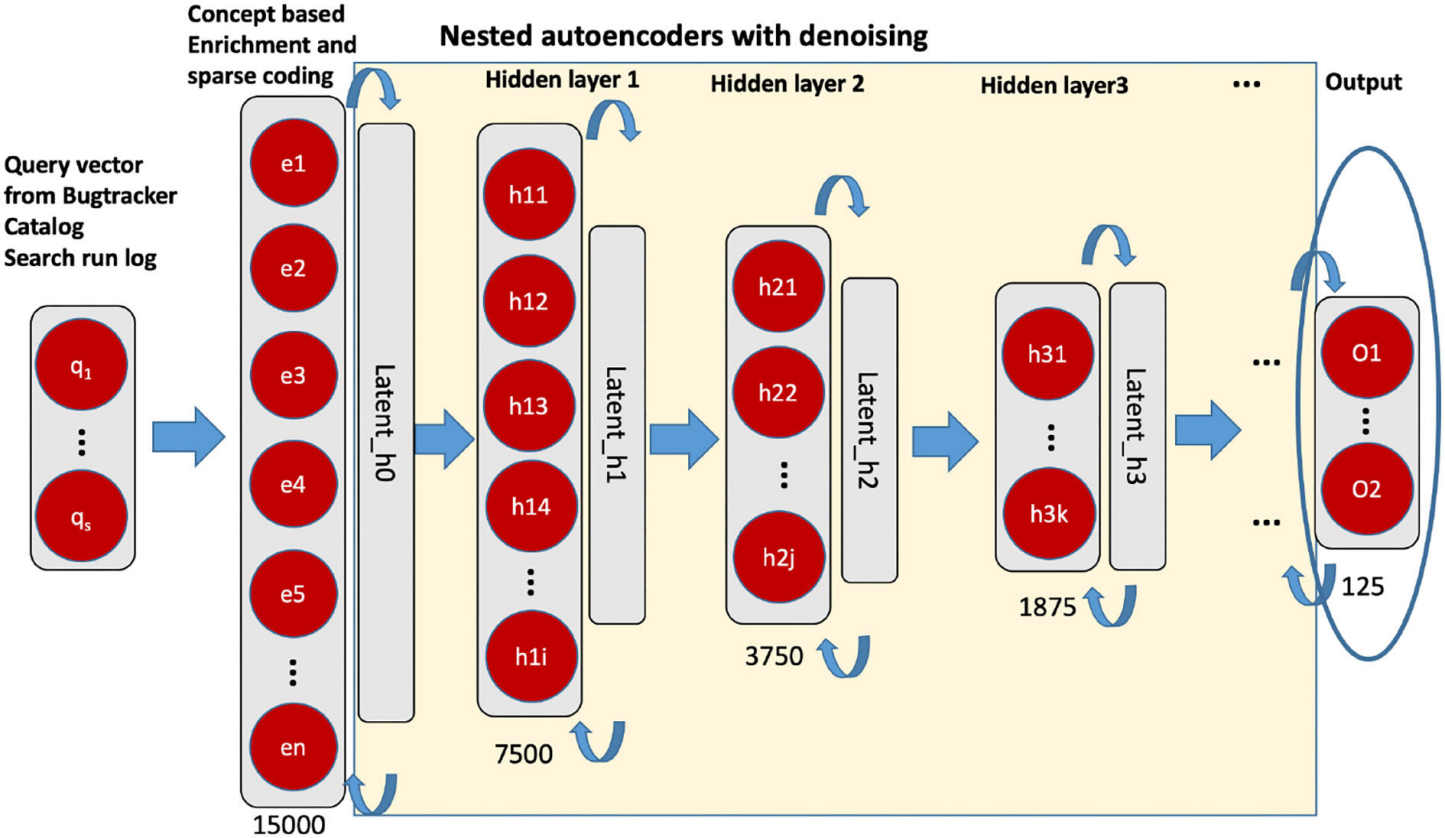
Unsupervised nested denoising autoencoders. The layer in circle is the representation vector (125 dimensions), we used for the downstream classification task.

After pre-processing, we conducted a dimension reduction as the second step. The principal component analysis (PCA) and stacked autoencoder have been used (see Figure 3). A PCA was applied to the vector representation of the 46,170 dimensional vector resulting in a 15,000 dimension vector. We determined this size as the output of the PCA according to the available hardware capacity and computing efficiency. As next, we applied a stacked denoising autoencoder for further representation learning. The stacked autoencoder is a layerwise non-linear method based on nested multiple two-layer autoencoders. We have followed the principle of layer-wise pre-training (unsupervised) so that a representation with better generalizability and differentiability can be obtained (for more details see Deng et al., 2018). After the dimension reduction with autoencoder, a dense vector with 125 dimensions is available as an input vector. The *i*^*th*^ query vector is defined as *q*_*i*_ = (*x*_1_, *x*_2_, ⋯ , *x*_*n*_)∈ℝ, where *n* = 125.

Generally, the attention mechanism enables the system to focus on specific subsets of the input (Vaswani et al., 2017). The selection of the subset is typically based on the state of the system which is itself a function of the previously attended subsets.

The goal of applying the attention mechanism in our settings is to let the network focus on specific aspects of the input and, thus, improve its ability to consider the most relevant information for all relevant inputs, thus yielding improvements in the quality of the generated hierarchical outputs.

**Figure 4:**
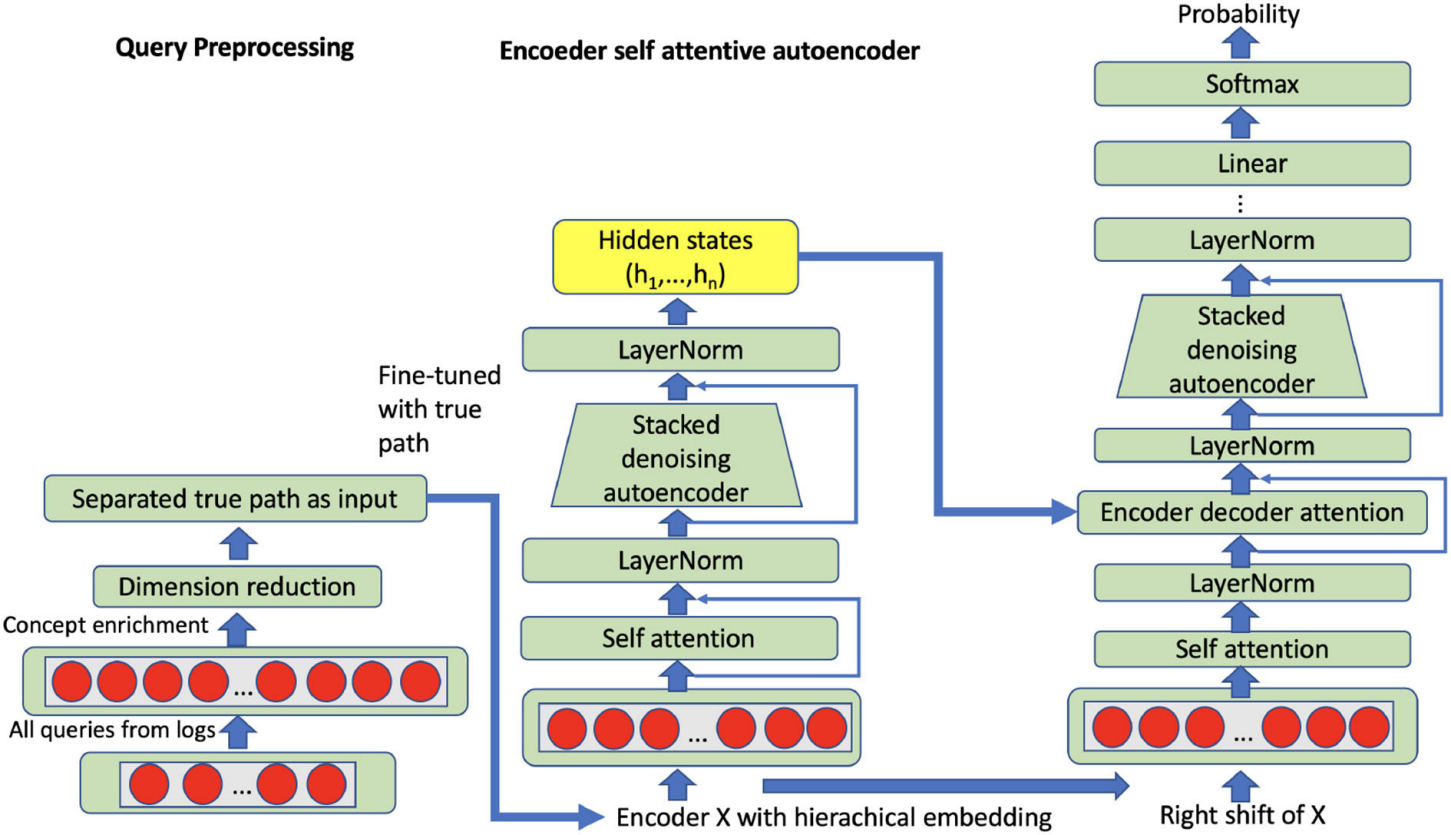
Self attentive autoencoder: The input can be all queries or grouped queries on all true paths.

The attention mechanism maps an input (the *q*_*i*_ vector with 125 dimensions) to a set of query-key-value pairs as output *Attention*(*Q, K, V*). The query is the entire representation on the true path; query (Q), key (K) and values (V) are obtained through the linear transformation with a learned parameter matrix based on input vector representation, while the three parameter matrices are the target of the self-attention mechanism. The output is computed as a weighted sum of the values, where the weight assigned to each value is computed based on the query and the corresponding key. In attentive autoencoders, there are three types of attention, namely encoding attention, encoder-decoder attention, and decoding attention (refer to Figure 4).


(9) $$\begin{array}{l} \text{Attention}\left(Q,K,V\right)=\text{softmax }\left(\frac{QK^{T}}{\sqrt{d_{k}}}\right)\;V \end{array}$$

where $Q\in {\mathbb{R}}^{n_{q}\times d_{k}}$, attentive query representation, generated based on the previous decoder, the multihead attention mechanism produces n subvectors from query input, which stack the query vector *q* into query matrix *Q*. *d*_*k*_ is the dimension of query vector and key vector, i.e., query and key have the same dimension. *d*_*v*_ is the dimension of the value vector. $K\in {\mathbb{R}}^{n_{k}\times d_{k}}$ and $V\in {\mathbb{R}}^{n_{k}\times d_{v}}$ are queries, keys, and values, respectively. Key values are calculated based on the output of the encoder. *n*_*q*_ is the number of queries and *n*_*k*_ is the number of key-value pairs. The encoder transforms a query into a list of vectors, one vector per input head. Given the input embedding sequence *x* = (*x*_1_, …, *x*_*n*_), we produce hidden representations, *h*_*e*_ = (*h*_*e*1_, …, *h*_*en*_) with the following equations:


(10) $$x=\left(x_{1},\ldots ,x_{n}\right)$$

(11) $$h_{e}=\left(h_{e1},\ldots ,h_{en}\right)$$

(12) $$a_{e}^{\prime }=\text{ Attention }\left(xW_{e}^{q},xW_{e}^{k},xW_{e}^{v}\right)$$

(13) $$a_{e}=LayerNorm\left(a_{e}^{\prime }+x\right)$$

(14) $$h_{e}^{\prime }=ReLu\left(a_{e}W_{e1}+b_{e1}\right)W_{e2}+b_{e2}$$

(15) $$h_{e}=LayerNorm\left(h_{e}^{\prime }+a_{e}\right)$$

where $W_{e}^{q}\in {\mathbb{R}}^{d_{m}\times d_{k}}$, $W_{e}^{k}\in {\mathbb{R}}^{d_{m}\times d_{k}}$, $W_{e}^{v}\in {\mathbb{R}}^{d_{m}\times d_{e}}$, $W_{e1}\in {\mathbb{R}}^{d_{m}\times d_{f}}$, and $W_{e2}\in {\mathbb{R}}^{d_{f}\times d_{m}}$ are parameter matrices; $b_{e1}\in {\mathbb{R}}^{d_{f}}$ and $b_{e2}\in {\mathbb{R}}^{d_{m}}$ are bias vectors. LayerNorm denotes layer normalization and ReLU is employed as the activation function. In order to compare with the aforementioned stacked denoising autoencoder, the same enriched query vector (15,000 dimensions) with external knowledge will be used as input for the self-attentive autoencoder. Besides, we have selected and grouped the queries for all possible true paths in our trainable nodes, so that the representation can be learned from all queries within full true paths. Particularly, we have 1,416 trainable true paths in total after we merge the CHOP and OPS data. Hence, we have trained 1,416 models based on a self-attentive autoencoder. After unsupervised self-attentive learning, the models have been fine-tuned through the task of supervised code classification. In the phase of code prediction, the corresponding model from self-attentive autoencoder and code classification can be called sequentially regarding the CHOP hierarchy (details refer to Section 5). For instance, the CHOP codes 75, 75.5, and 75.51 have 910, 681, and 131 positive queries, respectively. For this true path, we can train a self-attentive autoencoder with 1,722 training examples.

In contrast to the attention autoencoder method proposed by Zhang and Wu (2018), our self-attention component in the first transduction model has been extended with the queries from all specific true paths, refer to Figure 4. The residual connection has been used to connect the sub-layers.

The motivation for the application of self-attention is the length of the path and the construction of representation based on the unified vocabulary of the true path. Learning long-range dependencies is a crucial challenge in many sequence transduction tasks. It is noteworthy that the application of the deconvolution layer together with the transformer has also yielded enhanced results by image classification (Ruifrok and Johnston, 2001; He et al., 2022). The deconvolution layer upsamples the feature input and generates more sparser encoding and brings additional generalizability to the input with a more completely reconstructed target distribution. However, due to our preprocessing through the terminology server, the maximal generalizability namely the entire vocabulary has already been employed to enrich the input, our focus on learning and inferencing is, therefore, the exploring of correlation between those vocabularies. Hence, we have chosen a self-attention layer to cope with the transformer to achieve this all to all correlation learning instead of further upsampling through deconv-layer.

### 6.3. Training of base classifier using the hierarchical training set

For the selection of base classifiers, we have chosen random forest, Ada booster, and feed-forward neural network (DNN) with softmax. The random forest has proven to achieve both, high accuracy and efficiency as a base classifier (Atutxa et al., 2018), whereas the DNN and Ada booster have not been evaluated in previous studies. The stacked autoencoder and DNN have been implemented with Tensorflow. The random forest and the Ada booster have been realized with Scikit Learn 0.20.1 and Scipy 1.2.0. The DNN has two hidden layers (90–60) with 0.1 dropouts and AdamOptimizer. The random forest and Ada booster are both applied with standard configurations given by Scikit learn. Our neural networks related experiments have been performed on an NVIDIA DGX station (4 x Tesla V100, 256G main memory).

**Figure 5:**
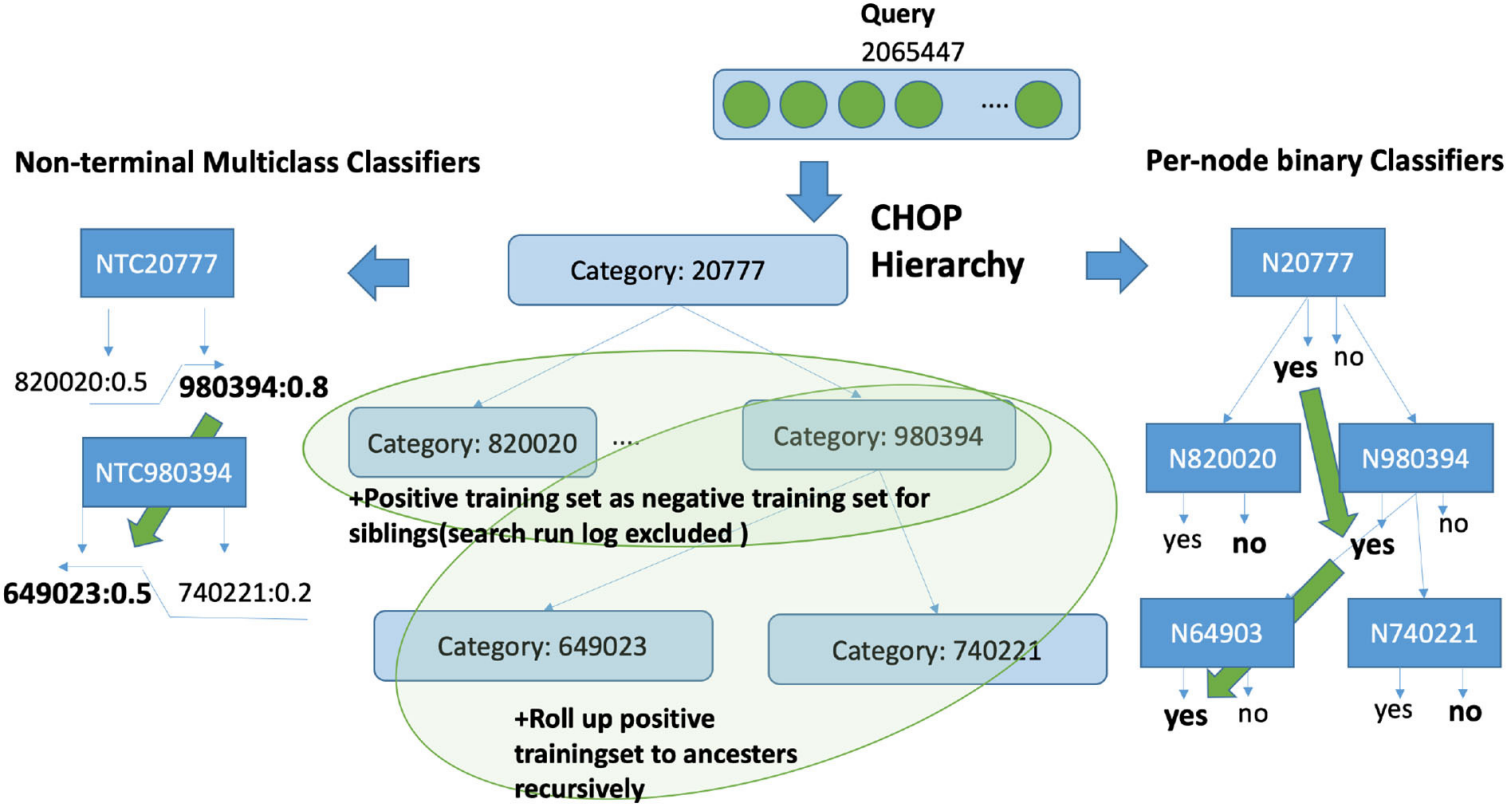
Hierarchical search based on non-terminal nodes and leaf nodes. The target query representation is learned with all query examples on its true path, so that the representation can be adapted according to all its relevant representations.

As already mentioned in Section 5, despite the pre-filtering, the positive and negative data sets are still strongly imbalanced. Hence, we have applied the oversampling method on the minority classes, while the majority class (negative examples) is kept the same (see Figure 5). We used the synthetic minority over-sampling technique (SMOTE) (Chawla et al., 2002), which generates new samples based on the distribution of existing samples. This method increases the proportion of positive examples and provides additional diversity to the training data set. With one trivial classification task against 8 top-level categories, we have validated the enhancement of accuracy after SMOTE oversampling, the 8 categories, classifications have not been improved by 3–4% within cross-validation. Considering this improvement, we have applied SMOTE oversampling on our entire data set and used the balanced data set as default. Hence, all the follow-up implementations are based on this enhancement through SMOTE oversampling.

### 6.4. Predicting the encoding path using base classifiers

**Figure 6:**
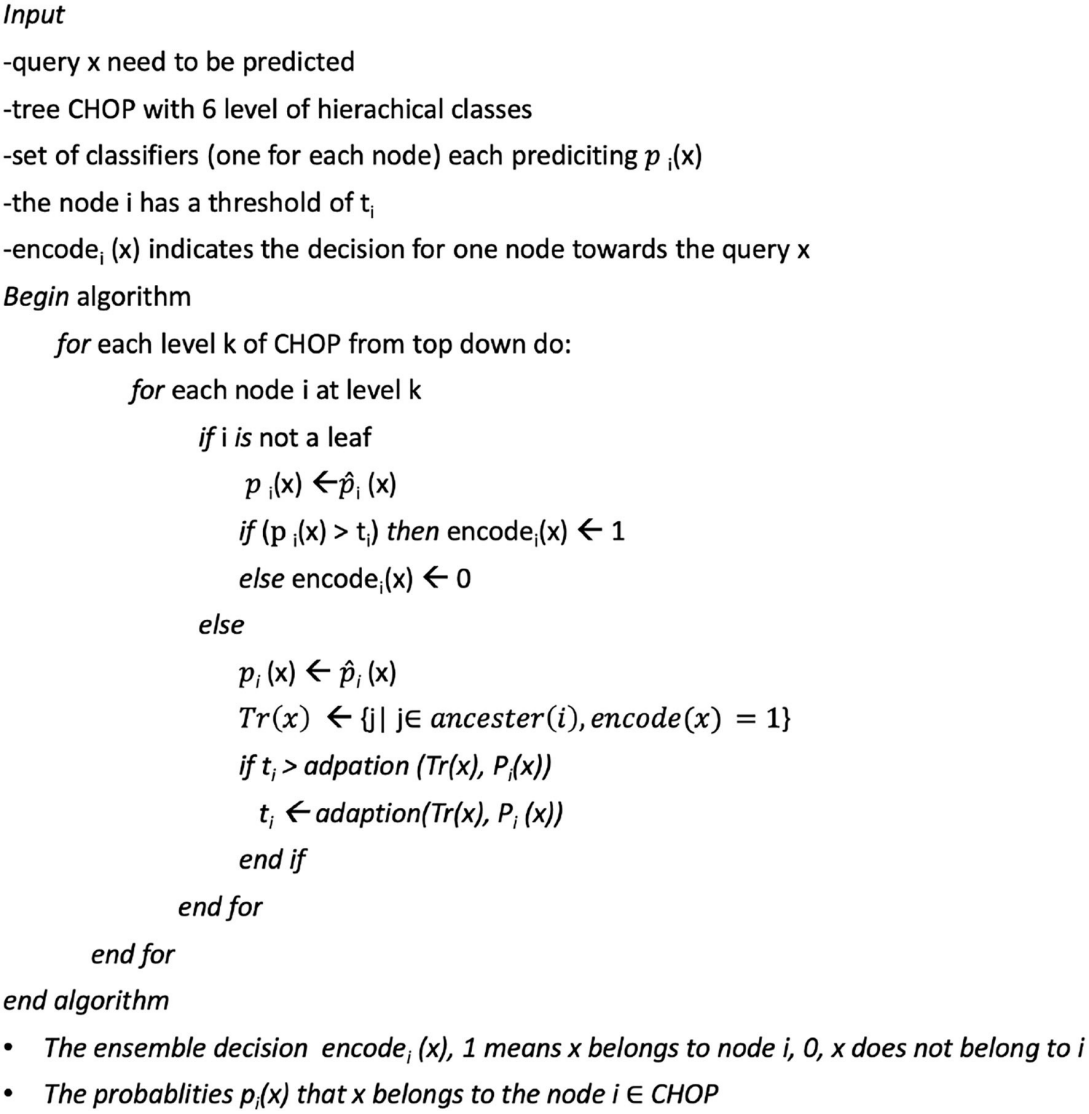
Algorithm of the hierarchical prediction of CHOP codes with base classifier. Adaptions refers to bottom-up average and Bayesian aggregation. More details about these two adaption methods are introduced in Section 6.5.

For the per-node binary classification (refer to pseudo-code in Figure 6), the search procedure starts from top-level categories (level 1). Seventeen top-level classifiers (one of the 18 categories has no training data) are called to determine the top category. After that, only classifiers from the relevant subcategories are invoked. The prediction procedure goes through the entire hierarchy recursively to level 6 with the binary results returned by the corresponding base classifiers. Let *encode*_*i*_(*x*) be a vector that stores the id of a prediction result for classifier x at level i. With a prediction call with n classifiers at one level, the decision *encode*_*i*_(*x*) is assigned to the predictor with the largest probability $encode_{i}\left(x\right)=argmax\left(\left[\hat{p_{1}}\left(x\right),\hat{p_{2}}\left(x\right),\cdots \;,\hat{p_{n}}\left(x\right)\right]\right)$, where the $\hat{p_{n}}$ represents the prediction result obtained by classifier n. Naturally, the prediction result can also be determined based on threshold t instead of maximal value. If $\hat{p}\left(x\right)>$ threshold *t*, the corresponding dimension of *encode*_*i*_(*x*) will be assigned with 1, otherwise with 0. A default setting for *t* is 0.5. The search result of level i is the sorted non-zero dimension of *encode*_*i*_(*x*). Similarly, the search using non-terminal classifier $\hat{p_{nt}}\left(x\right)$ proceeds top-down starting from the root recursively. When the classification of a query text reaches a non-terminal classifier, it will provide the most probable children category (pointer to the corresponding classifier) as the next classifier that should be called. The classifier of a category returns a score for each direct subcategory. However, the predictions $\left[\hat{p_{1}}\left(x\right),\cdots \;,\hat{p_{n}}\left(x\right)\right]$ are generated by one non-terminal multiclass classifier $\hat{p_{nt}}\left(x\right)$. Then, from the subcategories, the one with the highest score is chosen. The search proceeds recursively from that subcategory until a leaf category is reached. The last reached node in the hierarchy and its ancestor path is returned as the candidate category. Using this prediction method, we can find the single most suitable code for one query.

### 6.5. Hierarchical threshold adaption using bottom-up average and Bayesian aggregation

The prediction threshold refers to the probability boundary of predicted positive and negative labels. The setting of prediction thresholds by the classifier can fine-tune the performance of the prediction according to a hierarchical context. We adapt the smooth method mentioned in Notaro's work on the prediction within the human gene ontology (Notaro et al., 2017). They adapted the non-terminal node related threshold in a bottom-up way after the validation phase.

**Figure 7:**
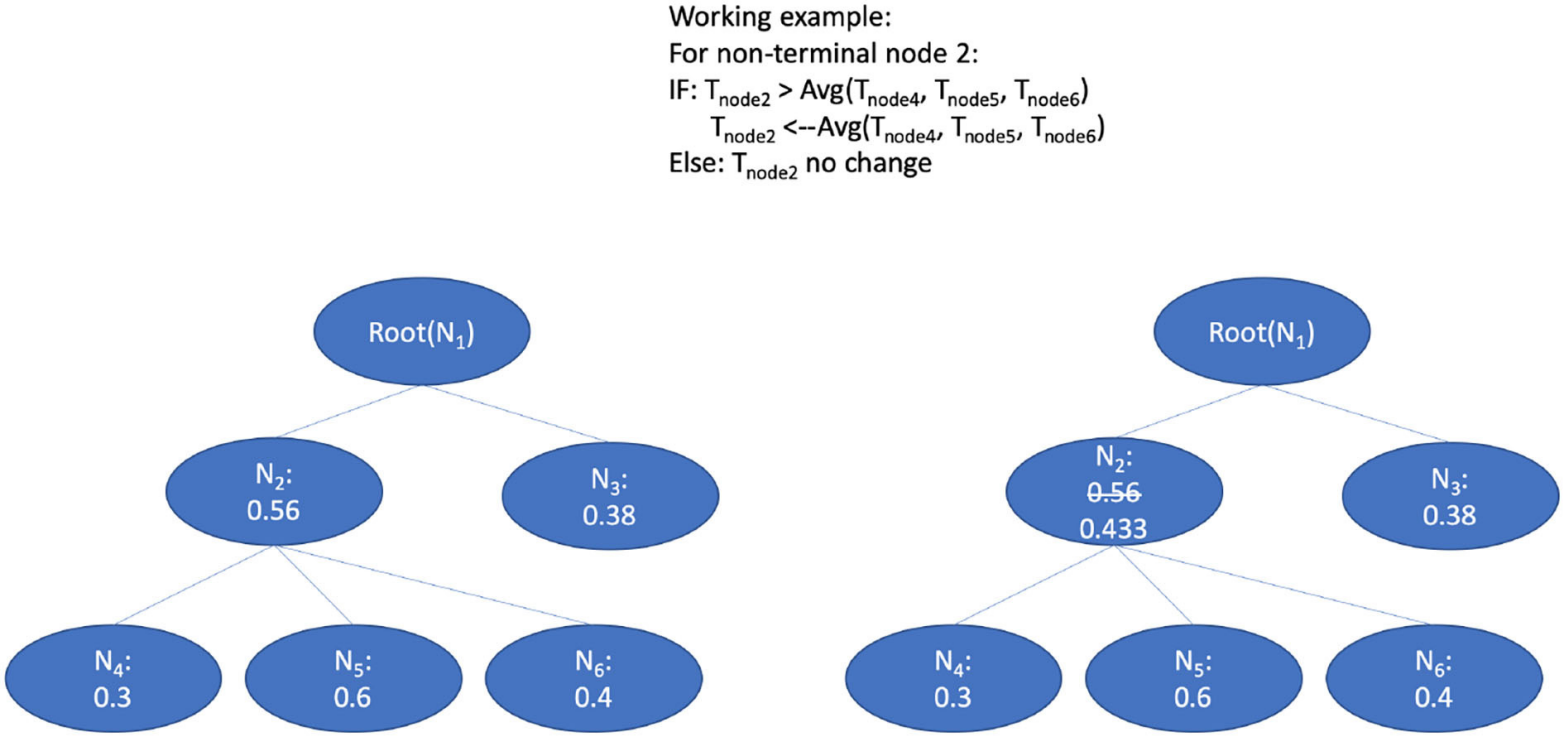
The bottom-up average for the threshold adaption.

The principle of the smooth process goes from leaf nodes to ancestor nodes. The threshold of the ancestor node will be compared with the average threshold value of all children nodes. Figure 7 shows the bottom-up strategy: 1) Use the smaller predicted value from the children nodes as a threshold to replace the threshold of their parent nodes recursively. 2) Values of the threshold for each node are initialized by the proportion of the positive and negative training data ($V_{threshold}=\frac{num_{positive}}{num_{positive}+num_{negative}}$) by the n-fold training and validation phrase; and 3) the threshold lowering aims to avoid the blocking of the true path since we observed that the node at the lower level (leaf level) can sometimes be blocked at the upper-level (in the direction of top-level) nodes. Thus, if the threshold value of the ancestor nodes is larger than the average threshold value from all children, the ancestor threshold will be replaced with this small value. Through this updating with the average values, the lower threshold value from children nodes will be propagated to upper-level nodes, which eases recognition of the true path regarding the thresholds. According to the evaluation results on a gold standard test, the per node threshold with smoothing done by children-node average thresholds has achieved a better augmentation in contrast to the settings with static thresholds (for details refer to Section 7.3).

**Figure 8:**
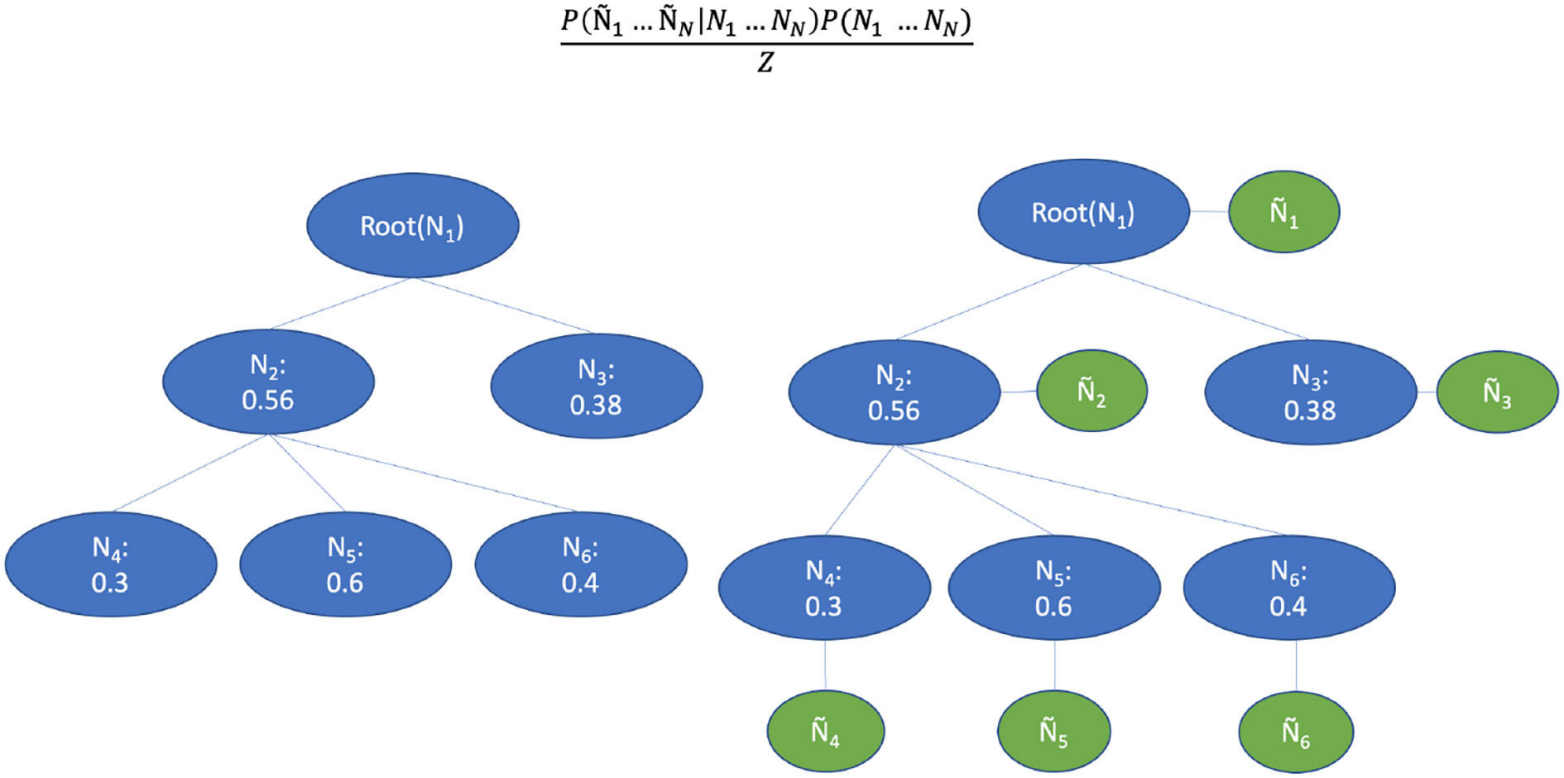
Bayesian aggregation for the threshold adaption. The blue circles represent the binary values of a CHOP node. The green circles represent the observed classifier outputs.

Inspired by the Bayesian aggregation method proposed by DeCoro et al. (2007), we used the classification results of a base classifier to construct the supportive Bayesian network, which represents the CHOP code structure. As can be seen in the right part of Figure 8, the variables were represented by a CHOP node in the hierarchy while the edges indicate conditional dependence. Let *y*_*i*_ represent the labeled binary outcome to class i, and ŷ_*i*_ represents the predicted value. The ${\vec{y}}_{{\text{parents}}_{i}}$ denotes the explicit affiliation to parent classes of i. E.g., let *y*_*i*_ stand for a category related to “computer assisted surgery based on MRI,” with ŷ_*i*_ the prediction of that procedure category, and ${\vec{y}}_{{\text{parents}}_{i}}$ representing the category of "computer assisted surgery with images support" and "computer assisted surgery" (top-levels). For n nodes in CHOP, the labels *y*_1_…*y*_*n*_ that maximizes the conditional probability need to be found through training and validation. *P*(*y*_1_…*y*_*n*_|ŷ_1_…ŷ_*n*_), which by Bayes rule equals


(16) $$\frac{P\left({\hat{y}}_{1}\ldots {\hat{y}}_{n}|y_{1}\ldots y_{n}\right)P\left(y_{1}\ldots y_{n}\right)}{D}$$

where D is a constant normalization factor. The class hierarchy shown on the left in Figure 8 is reconstructed into a Bayesian network by adding additional nodes that correspond to the observed classifier outputs. The h nodes are probability dependent on their parent classes and the ŷ-nodes are probability dependent on their corresponding labels h. The edges encode the conditional dependencies $P\left(y_{i}|{\vec{y}}_{\text{parents}\left(i\right)}\right)$, where ${\vec{y}}_{\text{parents}\left(i\right)}$ is used to denote all parent y-nodes of node *y*_*i*_. The true path $P\left(y_{i}|{\vec{y}}_{\text{parents}\left(i\right)}=1\right)$ is obtained from the training pairs. If the parent node of one label is irrelevant to the query, the label must also be irrelevant.


(17) $$\begin{array}{l} P\left(y_{1}\ldots y_{n}\right)=\overset{n}{\underset{i=1}{\prod }}P\left(y_{i}|{\vec{y}}_{\text{parents}\left(i\right)}\right) \end{array}$$

Since the CHOP has a tree structure in terms of code organization, the connection between *y* and ŷ represents the observation for a concrete encoding example on the CHOP path. Different from the reformulation proposed by DeCoro et al. (2007) under the independent conditions, our prediction result $\hat{y_{i}}$ is conditionally dependent on all other prediction $\hat{y_{i}}$ and labels *y*_*j*_ given true outcome *y*_*i*_. Then, we can reformulate the conditional probability as:


(18) $$P\left({\hat{y}}_{1}\ldots {\hat{y}}_{n}|y_{1}\ldots y_{n}\right)=\prod_{i=1}^{n}P\left({\hat{y}}_{i}|y_{i}\right)$$

The distribution of prediction *P*(ŷ_*i*_|*y*_*i*_) generated by base classifiers includes two variants that correspond to the binary classes of code matching [*P*(ŷ_*i*_|*y*_*i*_ = 1) and *P*(ŷ_*i*_|*y*_*i*_ = 0)]. The parameters can be estimated through cross-validation during the evaluation phase. By prediction, the query of the CHOP encoding will firstly be classified in a top-down manner using the base classifier. Finally, the trained Bayesian network can subsequently find the hidden *y* labels for the given ŷ predictions and fill the result to the decision set *encode*_*i*_(*x*).

## 7. Evaluation results

Different classification strategies were tested in the evaluation phase. The selection of base classifiers and trainable nodes is crucial for the performance of hierarchical classification. In general, the hierarchical prediction depends on the results of base classifiers. To determine the type of base classifier, we have performed experiments for both multi-class non-terminal nodes classification and per-node binary classification (one vs. rest) for 2,793 most frequently used procedure codes. After the comparison between multi-class non-terminal and per-node binary, we have extended the evaluation of per-node binary to 3,545 nodes based on queries from CHOP and OPS. The 2,793 (3,545 for OPS+CHOP) most frequent categories were obtained through the required number of positive samples with 21. A training set for each CHOP node with more than 21 positive examples ensures the minimal data requirement of the data balancing algorithm in the n-fold validation.

Subsequently, the 289 newly emerging queries (out of a sample set with 217 CHOP codes level 1 to level 6) are used to test the performance of code prediction. Different configurations with stacked denoising autoencoder, self-attentive autoencoder, and two threshold adaptions (average bottom-up and Bayesian aggregation) are evaluated.

### 7.1. Comparison of base classifier for non-terminal classifier and per-node classifier

**Figure 9:**
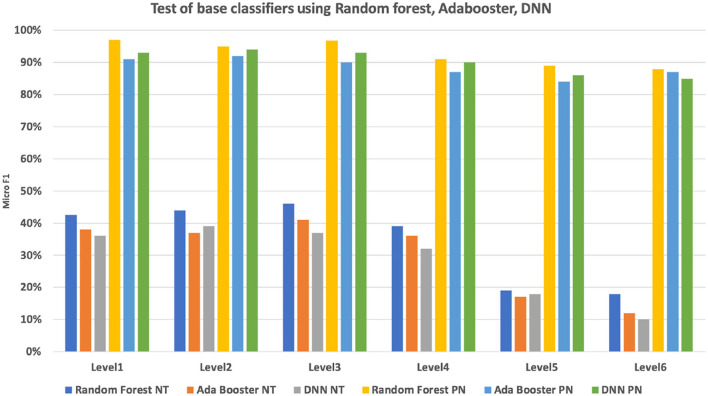
Micro F1 measure of multi-class non-terminal (NT) classifiers and per node (PN) ensembled binary classifier using random forest, Adabooster, and feedforward neural networks (DNN).

To evaluate the performance of the base classifier, we have conducted a base classification on the 2,793 nodes. The data set has been separated into 60% (2,947,308) training set, 40% (1,964,872) validation set for a 5-fold validation. We compared two strategies for base classifier training: non-terminal based classification (multiple classes output) and per node base classification (one vs. all classification). The performances for each of the six CHOP hierarchical levels were compared. It can be seen in Figure 9, the per node classifier has apparently outperformed the non-terminal classifier at each level, while the non-terminal classification achieves an F1 micro value between 39 and 46%. The per node classification results in an F1 micro value between 92 and 94%. Since we need to provide a product level implementation for the daily clinical usage to supplement the current system, we have only applied the random forest classifier with per node classification to conduct the follow-up hierarchical prediction.

### 7.2. Comparison between CHOP corpus and OPS training set

Based on the data in Table 1, the total training pairs have increased from 4,912,186 to 12,818,997, while the vocabularies have been augmented from 46,169 to 53,120. The trainable nodes in CHOP have therefore been increased from 2,793 to 3,545. Most of the logs that have been added are obtained from bug trackers and search logs from OPS DE (data obtained from the logging system for OPS DE code retrieval by ID MACS). As can be seen in Tables 2, 3, transferring OPS data to extend the CHOP corpus led to a decrease in the in-sample performance. The recall and precision were reduced by 2–9%.

**Table 2:** Trainable models regarding CHOP and performance of true code recognition based on pre-separated CHOP goldstandard test data.

**Level**	**Trainable nodes with CHOP corpus**	**Avg precision**	**Avg recall**	**Avg F1**
1	17	94.42%	90.89%	94.74%
2	104	93.12%	91.3%	92.20%
3	748	89.44%	85.36%	87.35%
4	958	91.3%	88.42%	89.84%
5	362	95.42%	89.23%	92.22%
6	45	93.52%	84.47%	88.76%

**Table 3:** Trainable models regarding CHOP plus OPS DE and performance of true code recognition on pre-separated CHOP goldstandard test data.

**Level**	**Trainable nodes with CHOP+OPS corpus**	**Avg precision**	**Avg recall**	**Avg F1**
1	17	90.34%	94.66%	92.45%
2	104	85.34%	89.44%	87.34%
3	793	89.57%	91.53%	90.54%
4	1,416	85.55%	87.43%	86.48%
5	1,030	81.43%	85.09%	83.22%
6	185	84.96%	90.47%	87.63%

### 7.3. Evaluation of the hierarchical catalogs of CHOP classification

The evaluation of hierarchical classification depends not only on the aforementioned in sample precision, recall, and F1 measure but also on out-of-sample performance using hierarchical metrics. The hierarchical features within CHOP will also be considered in our further evaluation so that the nature of CHOP encoding can be reflected. More specifically, the hierarchical precision represents the percentage of correct steps in the prediction. It punishes the incorrect steps. A match should not only be determined between one query and a single node but also the entire true path ranging from the retrieved root node to down-stream nodes. The hierarchical precision indicates the percentage of correctly predicted nodes along the true path, which punishes the missed nodes, while the hierarchical recall can be considered as the percentage of correctly predicted nodes, the missed corrected true paths (series of nodes) are punished.

**Figure 10:**
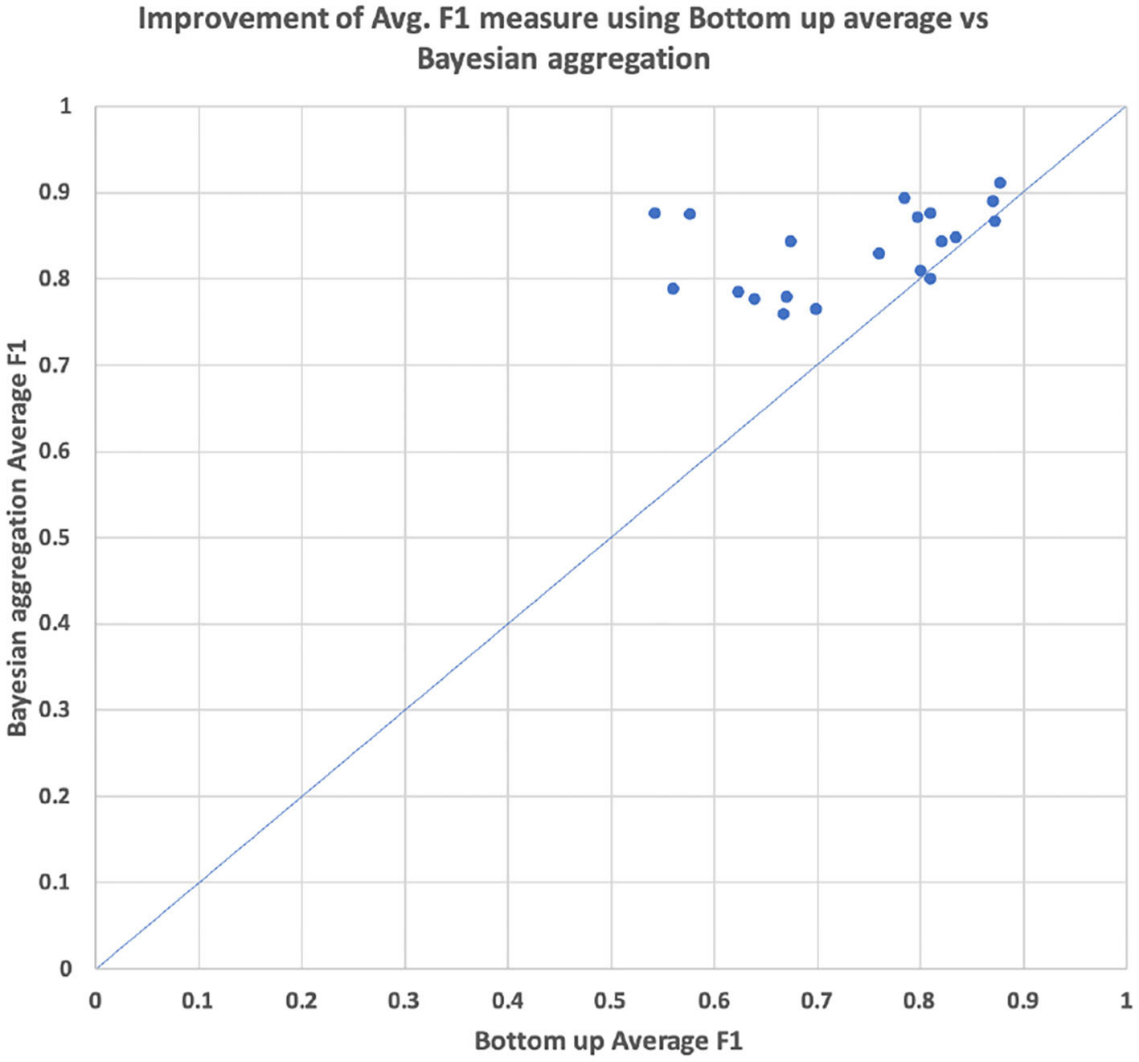
Comparison of performance average hierarchical F1 between bottom-up average threshold adaption vs. the threshold adaption with Bayesian aggregation based on 20 selected CHOP code from out of sample test sets.

As can be seen in Table 4, the hierarchical prediction based on the transferred data set (CHOP+OPS) has outperformed the prediction with the only CHOP data set. The recall has been particularly augmented through learning data transferring. The self-attentive autoencoder is one further optimization of the aforementioned stacked denoising autoencoder (Deng et al., 2018). The out-of-sample performances have been improved by self-attention for 6%, whereas the Bayesian aggregation offered another enhancement of 2% for average F1 in contrast to the adaption with average bottom-up. With both hierarchical self-attentive autoencoder and Bayesian aggregation, we have achieved the best F1 measure of 83.57%. In addition, we have visualized the average F1 measure of the prediction based on bottom-up adaption and Bayesian aggregation using 20 selected CHOP codes in our out-of-sample test set. The average improvements of the F1 measure for each code through both adaption methods have been illustrated through scattered points within the graph (refer to Figure 10). We selected the 20 codes with at least three positive query examples since we want to focus on the evaluation of average improvement on those codes with different query inputs instead of the codes with only one positive example or only negative examples. The Bayesian aggregation achieved a better average F1 measure than the average bottom-up adaption. The F1 values (scattered points in the graph) of Bayesian aggregation have generally located above the diagonal line in the graph, which presents better F1 performance in contrast to average bottom-up adaption. One further experiment presented in Table 5 has been performed based on the reorganization of the data set, the entire CHOP plus OPS DE data has been split into training 60/40%. In comparison with the previous setting of 70/30% splitting, the new split with 40% for the validation set has guaranteed that each node along the vertical path can get at least one test example. We have employed the success configuration with Bayesian aggregation (last line in Table 4 HA+CHOP+OPS+BAA), as is shown in Table 5, not all the trained nodes can be triggered except the top-level category. After the BAA and aggregation, the accuracy can be improved directly, particularly the last level (level 6), the BAA has yielded 40% of enhancement, whereas nearly 77% of the classifiers required a threshold adaption so that the CHOP codes can be generated in the corresponding level. One obvious trend is that the low level nodes (level 4 to level 6) require more threshold adaption in comparison with high level nodes (level 1 to level 3), which means the models for nodes at the lower level experienced more over-fitting and training bias due to lacks of data. However, the threshold adaption can be used to let the classification reach the low level nodes.

**Table 4:** Hierarchical performance comparison based on 289 newly emerging queries (out of a sample with 217 CHOP codes level 1 to level 6).

**Configuration**	**Avg precision**	**Avg recall**	**Avg F1**
Hierarchical prediction with SDA+CHOP	81.96%	60.33%	69.50%
Hierarchical prediction with SDA+CHOP+OPS	77.96%	69.43%	73.45%
Hierarchical prediction with ASA+CHOP+OPS	75.42%	74.23%	74.82%
Hierarchical prediction with HA+CHOP+OPS	80.96%	77.43%	79.16%
Hierarchical prediction with HA+CHOP+OPS+BUA	85.34%	74.53%	79.57%
Hierarchical prediction with HA+CHOP+OPS+BAA	86.96%	80.43%	83.57%

By default, top-down hierarchical prediction without thresholds adaption is applied. Stacked denoising autoencoder (SDA), All nodes self-attentive autoencoder (ASA), Hierarchical (true path selected), self-attentive autoencoder (HA), average bottom-up adaption (BUA), and Bayesian aggregation (BAA).

**Table 5:** Vertical reachability in CHOP plus OPS DE, triggerable classifiers before and after Bayesian aggregation and threshold adaption.

**Level**	**Classifier triggerable before/after adaption**	**Avg F1 before/after adaption**	**Percentage threshold adaption**
1	17/17	92.2/95.2%	0%
2	76/104	87.34/93.12%	21.4%
3	459/793	69.35/89.44%	35.36%
4	897/1,416	77.66/89.3%	67.42%
5	689/1,030	65.34/87.42%	89.23%
6	54/185	45.23/85.52%	77.47%

The validation set (40% of the data) was used to verify whether the trained classifier can be triggered correctly along the vertical path.

## 8. Discussion

The results clearly show that the per-node binary classification outperforms the non-terminal multiclass classification with an F1-micro measure between 92.6 and 94%. The hierarchical prediction based on per-node binary classifiers achieved a high exact match by the single code assignment on the 5-fold cross-validation.

With the purpose of data balancing as well as the increase of trainable nodes, we have transferred the German OPS data (similar medical procedure encoding used in Germany) as our input data. The number of positive training samples has therefore been supplemented. The data merging leads to a definite increase in the number of trainable nodes (3,545). However, the performance of the flat-classification decreased to 86–92% after the data merging. As the last step, the performance of models on a newly emerged data set has been increased from 80% (CHOP) to 87% (CHOP-OPS-merged), which indicates improved generalizability.

Different configurations have boosted the performance of the code prediction. The self-attentive autoencoder with all queries as input have outperformed the stacked autoencoder by 3%, while the input with selected queries on the true path has brought another 4% of improvement. The selection of input queries based on the CHOP hierarchy has clearly improved the precision of the task of code prediction and also offered a moderate improvement in recall. We believe that the query selection based on the true path has reduced the confounder between queries from sibling nodes and increased the connection among the features along with parent and child relations. The threshold adaption based on average bottom-up has slightly increased the F1 by 0.4%. The Bayesian aggregation has explicitly augmented the F1 for nearly 4%. The Bayesian aggregation has outperformed since the average bottom-up has only reflected the relation between two levels of node connection, whereas the Bayesian method has covered the entire true path in CHOP.

## 9. Conclusion and outlooks

In this paper, we conducted a hierarchical code assignment in the CHOP based on query text from server logs of an existing code retrieval system.

The stacked autoencoder with denoising function provided first a vectorized representation based on a unified vocabulary space for the query and CHOP catalog entries. Subsequently, an extension of the representation learning based on a self-attentive autoencoder has been applied to obtain a hierarchical representation with the same dimensional vector, which brings the hierarchical context into the compact representation. Using per-node training of classifier (random forest) and aggregation of training examples, we achieved high in-sample performance of code classification (94% micro avg F1).

The hierarchical prediction has been evaluated on the task of a single code assignment. More specifically, we simulated the path search with an adapted threshold of positive recall and average F1 of the base classifier. Based on the 289 newly emerging queries (out of sample), the proposed model has been tested. An F1 measure of 83.57% has been achieved with the configuration of a self-attentive autoencoder and Bayesian aggregation with both CHOP and OPS input data.

Through experiments, we can summarize the following answers to our research questions in Section 2. In general, the hierarchical context from the CHOP encoding can be employed by both classifier training and representation learning. The hierarchical features have all shown improvement in the classification performances under different configurations, respectively: the stacked autoencoder and training examples aggregation using true path rules as well as the unified vocabulary space have largely increased the utility of hierarchical features. By the hierarchical prediction, the possible solutions are bottom-up adaption and Bayesian aggregation. The bottom-up threshold adaption has increased the precision by 5% and the average F1 by 0.4%, while the Bayesian aggregation has even improved the average F1 measure by 4%.

Beyond that, the transfer learning using a training set from German OPS for training the CHOP classifier has increased positive examples and extended the number of trainable categories. The transfer of German OPS data led to an increase in accuracy of 4–5% in the task of prediction. As a next step, errors will be analyzed in more detail and the integration of the methods into the existing encoding system will be considered. The deployment of the model and selection of parameters set by the model application will be evaluated. Furthermore, the hierarchical labeling embedding (Wang et al., 2018) (vocabulary harmonization between query text and CHOP category) as well as threshold adaption with fuzzy matching (Lee et al., 2021) and the holistic optimization method can be evaluated.

## Data availability statement

Publicly available datasets were analyzed in this study. This data can be found the CHOP and OPS classification is available by the corresponding terminology provider (e.g., https://www.bfs.admin.ch/asset/en/18304268). Server logs cannot be provided.

## Author contributions

KD and YD: conceptualization, analysis of results, and writing. YD: methodology and conduction of experiments. Both authors have read and agreed to the submitted version of the manuscript.

## Conflict of interest

The authors declare that the research was conducted in the absence of any commercial or financial relationships that could be construed as a potential conflict of interest.

## Publisher's note

All claims expressed in this article are solely those of the authors and do not necessarily represent those of their affiliated organizations, or those of the publisher, the editors and the reviewers. Any product that may be evaluated in this article, or claim that may be made by its manufacturer, is not guaranteed or endorsed by the publisher.
